# Role of Cell Surface Hydrophobicity in the Pathogenesis of Medically-Significant Fungi

**DOI:** 10.3389/fcimb.2020.594973

**Published:** 2021-01-25

**Authors:** Carina Danchik, Arturo Casadevall

**Affiliations:** Department of Molecular Microbiology and Immunology, Bloomberg School of Public Health, Johns Hopkins University, Baltimore, MD, United States

**Keywords:** fungi, cell surface hydrophobicity, virulence, drug resistance, cell wall

## Abstract

Cell surface hydrophobicity (CSH) is an important cellular biophysical parameter which affects both cell-cell and cell-surface interactions. In dimorphic fungi, multiple factors including the temperature-induced shift between mold and yeast forms have strong effects on CSH with higher hydrophobicity more common at the lower temperatures conducive to filamentous cell growth. Some strains of *Cryptococcus neoformans* exhibit high CSH despite the presence of the hydrophilic capsule. Among individual yeast colonies from the same isolate, distinct morphologies can correspond to differences in CSH. These differences in CSH are frequently associated with altered virulence in medically-significant fungi and can impact the efficacy of antifungal therapies. The mechanisms for the maintenance of CSH in pathogenic fungi remain poorly understood, but an appreciation of this fundamental cellular parameter is important for understanding its contributions to such phenomena as biofilm formation and virulence.

## Introduction

Cell surface hydrophobicity (CSH) is a biophysical measurement of a cell’s affinity for a hydrophobic versus hydrophilic environment. Cells with higher CSH prefer a hydrophobic environment while those with lower CSH will preferentially remain in an aqueous environment (Krasowska and Sigler, 2014). This property can impact fungal virulence and biofilm formation (Galán-Ladero et al., 2013; Muadcheingka and Tantivitayakul, 2015; Dabiri et al., 2018) and is targeted by numerous antifungal drugs (Sivasankar et al., 2015; Kurakado et al., 2017; Suchodolski et al., 2020).

Despite the broad contributions of CSH to the biology and virulence of pathogenic dimorphic fungal species, data on this topic remains limited with most of the literature focusing on *Candida* species. A previous review of cell surface hydrophobicity in microbes (Krasowska and Sigler, 2014) also contained limited information on CSH in dimorphic fungal species, possibly because this area has not received as much interest in fungi as CSH in bacteria. However, given that CSH is a cellular property that has widespread effects on many aspects of microbial physiology, a more thorough understanding of fungal CSH is important, particularly as this virulence factor is altered by the switch between the yeast and hyphal forms of dimorphic fungi, which is essential for the virulence of certain pathogenic fungi (McBride et al., 2019; Sil, 2019; Staniszewska, 2020).

## Molecular Mechanisms of Hydrophobicity

CSH is a macroscopic property and extrapolating the molecular structures that determine it is difficult across the size scales. Nevertheless, the level of CSH must reflect the properties of the molecules on the surface of microbes that determine their interaction with water. Treatment of hydrophobic *Candida albicans* cells with proteases decreased CSH suggesting that this property was conferred by surface proteins (Hazen et al., 1990). However, these proteins are not always exposed. Depending on the growth condition they can be masked by hydrophilic fibrils (K. C. Hazen and Hazen, 1992) and the level of glycosylation (Hazen and Glee, 1994). Similarly, proteins appear to be responsible for CSH in *Aspergillus fumigatus* (Peñalver et al., 1996). In *Aspergillus* spp. these proteins include hydrophobins, small proteins with hydrophobic domains that allow interactions with hydrophobic surfaces (Wosten et al., 1993; Thau et al., 1994; Ohtaki et al., 2006). For *Cryptococcus neoformans* no information is currently available on the mechanisms responsible for CSH differences between strains or for the high CSH of some strains despite their surrounding hydrophilic polysaccharide capsule. The multifactorial nature of hydrophobicity presents a challenge in studying and understanding the impact of this property, especially in dimorphic fungi.

## Methods for Measurement of CSH

The microbial adhesion to hydrocarbons (MATH) assay is a common method for determining CSH in fungi (Borecká-Melkusová and Bujdáková, 2008; Ellepola et al., 2013a; Galán-Ladero et al., 2013; Rajkowska et al., 2015; Sivasankar et al., 2015; Ichikawa et al., 2017; Kurakado et al., 2017; Souza et al., 2018; Angiolella et al., 2020; Ramos et al., 2020; Suchodolski et al., 2020). This assay measures the decrease in culture density of an aqueous solution after thorough mixing with, and separation of, a hydrocarbon layer. Cells with low CSH will preferentially remain in the aqueous layer while cells with higher CSH will move into the hydrocarbon layer, decreasing culture density in the aqueous layer. Thus, a large decrease in aqueous culture density will occur in a sample with high CSH, and there will be minimal change in a sample with low CSH (Rosenberg, 1984). However, differences in protocols, settling cultures, and the inherent background noise of the assay can lead to variable results. This issue can be ameliorated by performing additional replicates (Ma et al., 2015) or using multiple methods to measure CSH in parallel (Ichikawa et al., 2017; Vij et al., 2020).

Another frequently used method quantifies the adherence of polystyrene microspheres to yeast cells (Antley and Hazen, 1988; San Millan et al., 1996; Hazen et al., 2000; Singleton et al., 2001; Ichikawa et al., 2017; Vij et al., 2020). Due to their hydrophobicity, the microspheres will attach selectively to hydrophobic cells. A cutoff of >3 beads per cell is widely used to designate hydrophobic versus non-hydrophobic yeasts, and individual cells can be counted and binned into these categories to determine the %CSH (Hazen and Hazen, 1987). Flow cytometry can also be used to separate these two populations when fluorescent microspheres are used (Colling et al., 2005).

For filamentous fungi, contact angle measurement can be used to determine the hydrophobicity of a mycelial mat although this technique is seldom used for human pathogens (Smits et al., 2003). In this method, drops of water are placed on top of the filamentous fungi mat and the contact angle between the water droplet and the mycelial mat is measured. A hydrophilic mycelial mat will have a low contact angle (cutoff of <30°) while a hydrophobic mat will have a higher contact angle (cutoff of >60°) (Smits et al., 2003).

## Factors Affecting CSH

Microbial CSH can be affected by multiple variables, including by altered cell wall composition, genetic modification, changes in temperature, and altered nutrient availability.

Among colonies of *Trichosporon asahii*, different yeast cell morphologies were indicative of differences in hydrophobicity (Ichikawa et al., 2017). Morphology is believed to affect virulence for this pathogen through an unknown mechanism. Colonies of several morphologies isolated from a single clinical sample had distinct morphologies that aligned with different levels of CSH. Heat-killing led to a slight but insignificant decrease in CSH for all strains, but when periodate was used to degrade cell wall polysaccharides, CSH dramatically decreased in all three strains tested. This suggests that the variance in cell wall composition and extracellular polysaccharides between strains of different morphologies may have directly contributed to the differences in CSH (Ichikawa et al., 2017).

Other components of the cell wall can also contribute to CSH in *C. albicans*, including mannoproteins, glucans, lipids, and chitin (Masuoka and Hazen, 1997; Pitarch et al., 2002). The sterol profile and lipid content also impact CSH in *C. albicans*, and differences in the expression of *ERG11* which encodes CYP51A1 alters both of these plasma membrane characteristics (Suchodolski et al., 2020). Additionally, knockout of *CSH1* which encodes a hydrophobic 38-kDa protein Csh1p decreased both CSH and adhesion to fibronectin in C. *albicans* (Singleton et al., 2001). Although a large portion of expressed Csh1p is localized to the cytoplasm, it is also associated with the cell wall and is upregulated upon increased temperature (Singleton and Hazen, 2004). Clearly a complicated property of the cell surface, CSH can be altered by manipulation of multiple components.

Nutrient availability, fungal growth phase, and temperature also affect CSH. Stationary phase cultures of *C. albicans* at 37°C diluted into fresh media can rapidly decrease their CSH in response to the newly accessible nutrients (Hazen and Hazen, 1988). Stationary phase cultures at either room temperature or 37˚C had an initial drop in CSH regardless of previous or current growth temperature, but CSH several hours after dilution was dependent on the growth temperature of the subculture with room temperature cultures having higher CSH (Hazen and Hazen, 1988).

In dimorphic fungi, the shift between hyphal and yeast forms is partially controlled by temperature and is associated with dramatic morphological, gene regulation, and cell surface composition changes. Most CSH studies in dimorphic fungi have focused on the organisms grown at 37°C (Muadcheingka and Tantivitayakul, 2015; Ichikawa et al., 2017; Llopis-Torregrosa et al., 2019; Angiolella et al., 2020; Ramos et al., 2020) although several studies have compared cultures grown at 37°C versus room temperature, identifying a general trend of higher CSH at lower temperatures and in the hyphal form (Antley and Hazen, 1988; Hazen and Hazen, 1988; Hazen et al., 1988; Singleton et al., 2001; Borecká-Melkusová and Bujdáková, 2008; Galán-Ladero et al., 2013).

Although most *Cryptococcus neoformans* are yeasts that usually do not have a filamentous form, they can expand from 5–7 μm to up to 100 μm and take on a distinct morphology known as titan or giant cells during infection (Zaragoza and Nielsen, 2013). In the context of *Galleria mellonella* infection, giant cells (>30 μm diameter) had similar infection outcomes to regularly sized *C. neoformans* despite being phagocytosed at a lower rate, demonstrating the importance of multiple virulence factors during infection. These giant cells had large cell bodies and thick polysaccharide capsules, both of which contributed to their increased size. The enlarged polysaccharide capsules additionally had decreased permeability. Although the study did not look at hydrophobicity, the reduction in capsule permeability suggests a structural change in the capsule which likely also alters the CSH (García-Rodas et al., 2011).

## Relationship to Biofilm Formation and Virulence

High CSH is generally considered to be a virulence factor for numerous dimorphic fungal species (Hazen et al., 1991). For the purposes of this discussion we will use the definitions of pathogenicity and virulence proposed by the damage-response framework of microbial pathogenesis whereby these refer to the inherent and relative capacities of microbes to cause damage in hosts, respectively, such that disease occurs if the damage is sufficient to affect homeostasis (Casadevall and Pirofski, 1999). Since the relationship between these properties and CSH involves a measurement of relative abilities, the term virulence is used. There are numerous studies in dimorphic fungi, especially on clinical isolates of *C. albicans* and non-*Candida albicans Candida* species, which demonstrate that high CSH is a common feature of these disease-causing isolates (Galán-Ladero et al., 2013; Muadcheingka and Tantivitayakul, 2015; Dabiri et al., 2018). Despite this, few studies have found any correlation between high CSH and biofilm formation, an important step in the establishment of infection, *in vitro*.

Adhesion is the process of cells attaching to another surface and is the first step of biofilm formation. CSH was directly correlated with adhesion across 12 clinical isolates of *Candida haemulonii* species complex. This correlation makes sense as CSH is a property of the cell surface which affects the way cells interact with their environment. Despite the substantial differences in CSH for these isolates and the correlation with adhesion for fungal cultures alone, no differences were seen between strains in a phagocytosis assay. Only one condition and time were tested, however, so it is possible that the kinetics would have varied at other time points or conditions and were simply not picked up in this assay. In a *Galleria melonella* infection model, only two strains had significantly more virulence, and both of these, surprisingly, had very low CSH (Ramos et al., 2020). In this instance, although CSH and adhesion were positively correlated, this did not translate to similar associations between CSH and phagocytosis or virulence in *Galleria melonella*.

Likely due to the many dynamics at play during the complex process of biofilm formation, high CSH does not have a consistent and direct association with biofilm formation ability. A comparison of CSH, adhesion to polystyrene, and biofilm formation ability for *Malassezia sympodialis* identified no significant correlation between CSH or adhesion and biofilm ability (Angiolella et al., 2020). This also held true for cells of several different morphology types from a clinical isolate of *Trichosporon asahii* which had no correlation between hydrophobicity and biofilm formation. In fact, the morphology associated with the highest CSH actually had the lowest ability to form biofilm (Ichikawa et al., 2017). An analysis of a hundred clinical isolates found a weak positive correlation was identified between CSH and biofilm formation ability for *C. albicans*, and a moderate correlation between these two factors was identified for *non-Candida albicans Candida* species (Muadcheingka and Tantivitayakul, 2015). Another study looked at CSH and biofilm formation ability across multiple *Candida* species and only found positive correlations between the two virulence factors for two of these species, *C. parapsilosis* and *C. tropicalis* while another found that adhesion and biofilm formation ability were moderately correlated with each other but neither was correlated with CSH (Dabiri et al., 2018; Souza et al., 2018).

The presence or absence of a relationship between CSH and biofilm formation may be temperature dependent. For *C. tropicalis* grown at 37°C, high CSH and filamentation, a transition from the yeast to hyphal state required for successful biofilm formation, were significantly correlated; however, no relationship was found between these two variables for the same strains grown at 22°C. Despite this association between CSH and filamentation at 37°C, neither CSH or adhesion correlated with biofilm formation, re-enforcing the understanding that these virulence factors are related but distinct (Galán-Ladero et al., 2013).

Additionally, higher CSH is not always correlated with higher virulence, as demonstrated by the *trk1Δ* mutant of *C. glabrata* (Llopis-Torregrosa et al., 2019). *TRK1* encodes for a high affinity potassium transporter which is the only potassium uptake system in this organism. Deletion of this gene impaired the cells’ ability to take up potassium and decreased cell fitness. The *trk1Δ* mutant also manifested altered the cell wall composition which, in turn, increased the CSH, regardless of potassium availability. The *trk1Δ* mutant formed significant biofilms at a wider range of potassium concentrations than the parental strain *in vitro*. Despite these *in vitro* results, in each of the three infection models tested (*Drosophila melanogaster*, *Galleria mellonella*, and THP-1 macrophage-like cells), the *trk1Δ* mutant was less virulent than the parental strain. In animal hosts, extracellular potassium is generally in the low mM range, and the mutant cells grew poorly under that condition. This overwhelmed the benefit of increased CSH, adherence, and biofilm formation in these pathogens because they were not able to grow well in the host environment and demonstrates that not only CSH itself, but also the mechanism of CSH, is important for virulence (Llopis-Torregrosa et al., 2019).

The host response to pathogen exposure can also alter CSH. During infection, pathogens may be bound by antibodies that coat their surfaces. Three mAbs (21E6, B9E, and 3D9) targeting cell wall fibrillar adhesins important for the adhesion of *C. albicans* germ tubes were tested for their effects on adhesion to polystyrene and filamentation (San Millan et al., 1996). mAb 21E6 enhanced adhesion but reduced filamentation, and mAb B9E decreased both adhesion and filamentation. mAb 21E6 decreased the CSH of C. albicans while B9E increased it. 3D9 had no reactivity against the tested adhesins, did not alter CSH, and had no effect on either process (San Millan et al., 1996). These effects were antibody-specific and may have different mechanisms. In *C. neoformans*, binding of the protective capsular mAb 18B7 increased CSH in a dose-dependent manner while two non-protective capsular mAb 12A1 and 13F1 had no effect on CSH (Vij et al., 2020). This increase in CSH will presumably facilitate engulfment by macrophages. Thus, antibody binding as a component of the immune response to infection may act in part through exogenous manipulation of CSH to promote pathogen clearance.

Biofilm formation and virulence are multifactorial processes, and CSH has a much more direct relationship with adhesion than with the more complex process of biofilm formation. Biofilm formation is often a key step in fungal pathogenesis and establishment of infection. Although CSH is not often directly correlated with biofilm formation, it may promote virulence through more complex and currently unexplored mechanisms which are not recapitulated under the *in vitro* conditions tested, resulting in higher CSH being associated stronger virulence.

## Response to Antifungal Treatments

An understanding of CSH is also critical when developing new antifungal strategies as differences in CSH can cause variable responses to antifungal treatment. In addition, many therapeutics reduce CSH. This emphasizes the importance of considering CSH when determining the best treatment option.

Fluconazole is a major antifungal drug, which inhibits ergosterol metabolism by blocking the activity of CYP51A1. One recent study found that fluconazole had much lower efficacy against a more hydrophobic strain of *C. albicans* (Suchodolski et al., 2020). Fluconazole was tested, alone or in combination with gentamicin, against two strains of *C. albicans*: CAF2-1 and CAF4-2. CAF4-2 had higher CSH as well as higher expression of *ERG11*, the gene which encodes for CYP51A1, and promotes ergosterol production and *CDR1* which encodes Cdr1, a drug efflux pump. Fluconazole treatment increased the CSH of the more hydrophilic CAF2-1 strain but not that of CAF4-2. It also increased *ERG11* expression much more in the hydrophilic than the hydrophobic strain, demonstrating that CYP51A1 overexpression is protective against fluconazole treatment (Suchodolski et al., 2020). This mechanism of resistance holds true across numerous studies and was previously reviewed (Berkow and Lockhart, 2017). *ERG11* overexpression can have multiple causes, including gain of function mutations in the transcription factor Upc2p which is induced upon ergosterol depletion, altered sterol biosynthesis, and mutations in *ERG11* itself (Heilmann et al., 2010; Berkow and Lockhart, 2017).

Differences in *ERG11* expression alter the surface sterol profile and presumably the CSH through this mechanism. Therefore, the difference in treatment response between the CAF2-1 and CAF4-2 strains may be due to changes in lipid homeostasis and metabolism (Suchodolski et al., 2020). Another older study, however, found that a subinhibitory concentration of fluconazole did not affect CSH in *C. albicans* although it did sensitize the fungi to killing by murine polymorphonuclear leukocytes (Hazen et al., 2000). This difference is likely due to the specific strains used in each study as, in the more recent study, fluconazole only affected CSH in one of the two strains tested.

Subinhibitory doses of four antifungals, including fluconazole, were tested for their effects on CSH and biofilm formation ability in both *C. albicans* and *C. dubliniensis* (Borecká-Melkusová and Bujdáková, 2008). The 50 isolates were classified into four genotypes based on the details of the presence or absence of a group I intron at a specific location on the 25S rRNA gene, three genotypes for *C. albicans* (A, B, and C) and one for *C. dubliniensis* (D). The *C. dubliniensis* isolates were generally more hydrophobic than the *C. albicans* ones. In the tested range of concentrations, fluconazole did not affect CSH of *C. albicans* but did effectively reduce biofilm formation. Conversely, it decreased CSH in *C. dubliniensis* but did not decrease biofilm formation. Voriconazole reduced both biofilm formation and CSH for all four genotypes tested. Amphotericin B decreased CSH in all genotypes but only reduced biofilm formation for genotypes A, B, and D. Itraconazole decreased CSH in genotypes A, B, and D and decreased biofilm formation for all three genotypes of *C. albicans*. Because the comparisons were made among aggregates of data for all four genotypes, some information may have been lost as each genotype contained a wide range of CSH values, but decreased CSH and biofilm formation were both common outcomes of low dose antifungal exposure, and the two appear to be independent of each other (Borecká-Melkusová and Bujdáková, 2008).

Chlorhexidine gluconate is a common active ingredient in mouthwash with broad antimicrobial activity. Even at subtherapeutic doses, it has been shown to decrease CSH in *C. dubliniensis* and *C. albicans* (Ellepola et al., 2013a; Ellepola et al., 2013b). As CSH helps to mediate adhesion which is necessary for the establishment of infection, chlorhexidine gluconate, even at low doses, may help to reduce oral fungal infection.

Minocycline, a tetracycline derivative, was active against the budded-to-hyphal-form transition of *C. albicans* at sub-growth inhibitory concentrations (Kurakado et al., 2017). This transition is necessary for full virulence and for biofilm formation. Minocycline significantly decreased CSH at the concentration necessary for decreased biofilm formation. In addition, it downregulated the expression of several hyphal and biofilm formation-related genes. These included the hypha-specific genes *HWP1* and *ECE1*, the hypha-related transcription factor genes *EFG1*, *CPH1*, and *TEC1*, the adhesion-related gene *ALS3*, and the biofilm-related gene *BCR1* (Kurakado et al., 2017).

*Malassezia* spp. have a high percentage of lipids in their cell wall (~15%–20% w/w) which contributes to their high CSH. L-glutathione, an antioxidant with antifungal activity, decreased CSH by 85%–95% in four species of *Malassezia* without affecting viability (Sivasankar et al., 2015). It increased the time to cell aggregation specifically through its reduction of CSH without altering cell surface charge as measured by zeta-potential. It also decreased fungal virulence in a blood sensitivity assay by 64%–73% for the same four species, suggesting that CSH is a major virulence factor for this genus of dimorphic fungi (Sivasankar et al., 2015).

The effects of numerous non-clinically approved agents on CSH have also been assessed in various dimorphic fungal species. The extract of the plant *Eugenia uniflora* which has antioxidant and antimicrobial activity was tested against *Candida* spp. and was able to decrease CSH, adhesion to human buccal epithelial cells, and biofilm formation to different extents (Souza et al., 2018). Several essential oils, specifically tea tree, thyme, and clove, have also been examined for their effects on CSH in several *Candida* species. The results were essential oil and strain-dependent, but, particularly when used in combination, the essential oils tested often significantly decreased CSH (Rajkowska et al., 2015). A bacteriocin isolated from *Streptococcus sanguinis* culture media was also effective against *C. albicans* and *C. tropicalis*. Although its activity was multifactorial, it decreased CSH (Ma et al., 2015). Reducing CSH appears to be a common activity for many antifungal agents, both clinically approved and investigational.

As may be anticipated, higher CSH is associated with higher phagocytic efficiency. Increasing CSH in *C. neoformans* was positively correlated with phagocytosis by the natural predator *Acanthamoeba castellani* (Vij et al., 2020). Additionally, in *C. albicans*, murine polymorphonuclear leukocytes (PMNs) were more effective at engulfing more hydrophobic cells cultured at room temperature than they were for cells grown at 37°C (Antley and Hazen, 1988). The opposite trend was seen for cell killing, however, with PMNs being more effective at killing the less hydrophobic cells grown at 37°C. This is due to the enhanced ability of the room temperature cultured cells to form germ tubes. Germ tube formation is correlated with CSH. In line with this, *C. albicans* grown at room temperature had higher CSH and led to more rapid death in a mouse model than *C. albicans* cells grown at 37°C (Antley and Hazen, 1988; Hazen et al., 2000). Therefore, the impact of CSH on phagocytosis and killing appears to be two-fold. Higher hydrophobicity makes the cells initially easier to engulf, but also makes them more prone to germ tube formation and resistance to phagocytic killing.

CSH is clearly an important virulence factor to consider in relation to both treatment options and drug development. As CSH can alter the efficacy of antifungal therapies, it could be helpful in informing treatment selection. CSH is also frequently reduced by antifungal therapies as part of their activity and provides a good, easily quantifiable phenotypic readout which can be used to measure this virulence factor *in vitro*. In addition, further exploration of the mechanisms through which these drugs reduce CSH and subsequently virulence could elucidate additional pathways involved in pathogenesis and lead to the development of novel strategies to target fungal infections.

## Synthesis

Our review of the CSH information available for medically-important fungi revealed disparate observations obtained from different organisms and variable experimental settings. This makes it difficult to propose a coherent perspective that includes all of these observations into on cohesive theory, especially given that some studies report conflicting results. Although this complicates a clear, mechanistic understanding of the impact of CSH on pathogenesis and drug resistance across organisms and studies, CSH is a property of the cell wall, which provides a protective barrier between the cell and its environment and as such, is indisputably a key factor in these processes. More work on this topic, particularly in other medically-significant dimorphic fungi such as *Coccidiodes immitis*, *Paracoccidioides brasiliensis*, *Blastomyces dermatitis*, *Histoplasma capsulatum*, and *Sporothrix schenckii* is needed because the literature on the CSH species is limited or non-existent. In this regard, comparative studies across diverse species could provide additional important insights on the specific effects of high or low CSH, which might help clarify the current, conflicting results. We are hopeful that our delineation of the variable effects of CSH on virulence, biofilm formation and drug resistance stimulates additional studies to explore how this critical cell parameter affects these processes.

## Conclusions

The body of work available on CSH in dimorphic fungi, mostly in *Candida* species, demonstrates that this biophysical parameter plays important and complex roles in the processes of virulence, biofilm formation, and response to treatment. High CSH frequently but not universally corresponds to stronger virulence. Although related, CSH is distinct from biofilm formation, and there is often no direct correlation between the two properties, although both are important for virulence. Much of the existing literature on CSH in dimorphic fungi focuses on *Candida* species, and similar experiments for other pathogenic dimorphic fungi could provide a better understanding of these organisms from both a basic science and clinical perspective. In general, CSH is a relatively understudied cellular property in fungi that merits more attention given its fundamental nature for microbial physiology, cellular attachment, virulence, and as a drug target.

## Author Contributions

CD wrote the manuscript. AC edited and wrote parts of the manuscript. All authors contributed to the article and approved the submitted version.

## Funding

AC was supported in part by NIH grants AI052733, AI15207, and HL059842. CD was supported by NIH grant GM008752.

## Conflict of Interest

The authors declare that the research was conducted in the absence of any commercial or financial relationships that could be construed as a potential conflict of interest.
